# Seed-Zone Salinity and Regional Ionic Signatures Associated with Cotton Emergence Failure Under Mulched Drip Irrigation in Southern Xinjiang

**DOI:** 10.3390/plants15192928

**Published:** 2026-09-24

**Authors:** Tong Qi, Jian Huang, Zhiguo Wang, Wanli Xu, Kelin Hu

**Affiliations:** 1 Institute of Agricultural Resources and Environment, Xinjiang Academy of Agricultural Sciences, Urumqi 830091, China; tong1982nn@163.com (T.Q.); huangjian@xaas.ac.cn (J.H.); wangzhiguo214@126.com (Z.W.); 2 College of Land Science and Technology, China Agricultural University, Beijing 100193, China

**Keywords:** cotton establishment, EC_1:5_, field diagnostic cut-point, paired sampling, saline soil, soluble ions, Xinjiang

## Abstract

Soil salinity can constrain cotton (*Gossypium hirsutum* L.) establishment in southern Xinjiang, where emergence may vary sharply within uniformly managed fields. We conducted a paired observational field survey of 130 commercial cotton fields, comparing well-established microsites (A; live-seedling stand ≥ 80%) with non-emerged or nearly non-emerged microsites (B; emergence < 5%) in Kashgar (90 pairs) and Aksu (40 pairs). Soil was sampled 14–28 d after sowing, after establishment status was evident. B microsites had higher total water-soluble salts (13.54 vs. 5.95 g kg^−1^), EC_1:5_ (2.98 vs. 1.21 mS cm^−1^), and SAR_1:5_ (4.17 vs. 1.32; all q < 0.001). Matched models showed that EC_1:5_ remained associated with the B class after adjustment for soil texture and either Na^+^ or Cl^−^. Direct pair-level tests confirmed regional differences in ionic-composition change (permutation *p* < 0.001), with relatively stronger Na^+^–Cl^−^ enrichment in Kashgar and Ca^2+^–SO_4_^2−^ enrichment in Aksu. Exploratory in-sample EC_1:5_ diagnostic cut-points were 2.51 mS cm^−1^ for Kashgar and 1.17 mS cm^−1^ for Aksu. Because sampling was post-outcome and the cut-points were internally derived from a contrast-selected dataset, causal interpretation and field application require longitudinal evidence and external validation.

## 1. Introduction

Soil salinization is a major constraint on irrigated agriculture in arid and semi-arid regions, where low precipitation, high evaporative demand, shallow groundwater, and repeated irrigation promote the accumulation of soluble salts in the upper soil profile [1,2,3]. Xinjiang is one of China’s major cotton-producing regions and contains extensive areas of salt-affected agricultural land [4,5]. Although cotton is generally considered moderately salt tolerant at later growth stages, with classical yield-response relationships based on saturated-paste electrical conductivity, germination and early seedling establishment are substantially more sensitive to salinity than mature crop growth [6,7,8,9]. Salinity during the relatively short emergence period may therefore strongly influence final stand density, even when subsequent irrigation partially leaches salts from the root zone.

Under plastic-mulched drip irrigation in southern Xinjiang, pronounced within-field differences in cotton emergence are frequently observed: cotton may emerge normally in one area but remain sparse or absent only tens of metres away despite the same cultivar, sowing date, irrigation system, and fertilization program. Previous studies in southern Xinjiang have documented substantial spatial variability in soil salinity under mulched drip irrigation and shown that emergence-irrigation practices can alter seed-zone water and salt conditions [10,11,12,13]. However, most previous irrigation experiments and field surveys have emphasized treatment means or field-scale salinity patterns. The specific advance of the present study is the use of within-field paired emerged/non-emerged seed-zone microsites across commercial fields to ask which soil properties distinguish contrasting emergence outcomes under shared field-level management. This paired diagnostic framework targets local contrasts that field-scale mapping or treatment-mean comparisons do not directly resolve.

Total salinity is an integrative measure of the soil salt environment because elevated dissolved-salt concentrations can reduce soil water potential and increase the energy required for plant water uptake. Ionic composition may additionally modify plant responses: Na^+^ and Cl^−^ can exert ion-specific effects on plant nutrition and cellular function, whereas a high proportion of Na^+^ relative to divalent cations may contribute to sodicity-related structural instability when exchangeable sodium is also elevated [14,15,16,17,18]. In contrast, Ca^2+^–SO_4_^2−^-rich salinity represents a chemically different soil–water environment from Na^+^–Cl^−^-rich salinity. These physiological and soil-chemical processes provide biological context for the field associations examined here; the present observational design does not experimentally separate osmotic stress from Na^+^- or Cl^−^-specific toxicity or directly test a mechanistic causal pathway. Because the major soluble ions are strongly correlated with total salinity, attributing emergence failure to a single “causal ion” on the basis of univariate field comparisons is statistically problematic. Paired multivariable and multivariate analyses are therefore needed to distinguish overall salinity associations from differences in ionic composition.

Soil physical properties may also influence cotton emergence and salt redistribution. Soil texture affects water retention, wetting-front geometry, evaporation, and the persistence of salts around the seed zone. Importantly, particle-size distribution is not equivalent to soil structural stability; a coarser texture measured at a non-emerged microsite cannot, by itself, demonstrate sodium-induced clay dispersion. Distinguishing pre-existing textural heterogeneity from salt-induced structural degradation requires additional measurements, such as exchangeable sodium percentage (ESP), cation exchange capacity (CEC), aggregate stability, and hydraulic conductivity, which are rarely available in regional field surveys. Related agricultural-soil studies have also emphasized water-balance evaluation in arid irrigated fields and broader physicochemical characterization of cultivated soils [19,20].

A within-field paired design provides a useful observational framework because each non-emerged microsite can be compared with a nearby successfully emerged microsite within the same field, thereby accounting for many field-level management factors by design. Accordingly, the objectives of this study were to: (1) quantify paired differences in seed-zone salinity, ionic composition, pH, and particle-size distribution; (2) determine whether EC_1:5_ and individual ions provide independent information in matched multivariable models; (3) identify common and region-specific ionic signatures using concentration-based PCA and complementary compositional analysis; and (4) derive exploratory field diagnostic cut-points using pair-preserving ROC analysis. We specified three testable hypotheses: (i) non-emerged microsites would have higher measured seed-zone salinity than their paired emerged microsites; (ii) EC_1:5_ would remain associated with non-emergence after accounting for soil texture and major ionic indicators such as Na^+^ or Cl^−^; and (iii) the within-pair change in ionic composition would differ between Kashgar and Aksu. Support for the third hypothesis required a statistically supported regional difference in pair-level ionic-composition change, rather than opposite PCA centroid directions alone. ROC analysis was treated as an exploratory analytical objective rather than a biological hypothesis.

## 2. Results

### 2.1. Paired Seed-Zone Soil Characteristic Contrasts

Across the 130 complete field pairs, non-emerged microsites had substantially greater salinity than adjacent emerged microsites (Table 1; Figure 1). Mean total salt increased from 5.95 ± 4.19 to 13.54 ± 7.00 g kg^−1^, and EC_1:5_ increased from 1.21 ± 0.75 to 2.98 ± 1.37 mS cm^−1^. Mean SAR_1:5_ was 1.32 ± 1.14 at A positions and 4.17 ± 3.36 at B positions. All three differences remained highly significant after FDR correction (q < 0.001). The paired pattern was highly concordant: B exceeded A in 95.4% of pairs for total salt, 97.7% for EC_1:5_, and 86.9% for SAR_1:5_.

Na^+^ and Cl^−^ showed the largest relative enrichments among the major ions. Mean Na^+^ increased from 0.276 to 1.542 g kg^−1^ (5.6-fold), and Cl^−^ from 0.460 to 2.174 g kg^−1^ (4.7-fold). Mg^2+^ increased 3.9-fold, whereas SO_4_^2−^ and Ca^2+^ increased 1.9- and 1.6-fold, respectively. Although Na^+^ was one of the strongest univariate indicators, its high correlation with EC_1:5_ and total salt indicated that its independent contribution required multivariable assessment.

Particle-size distributions also differed within fields. Emerged microsites had higher clay (9.01% vs. 6.60%) and silt (22.70% vs. 15.08%), whereas non-emerged microsites had higher fine sand (73.06% vs. 60.76%). Coarse sand did not differ significantly after FDR correction. These measurements describe intrinsic particle-size composition and do not by themselves demonstrate a change in aggregate structure.

### 2.2. Matched Multivariable Models

In the matched conditional logistic model containing EC_1:5_, clay, and silt, standardized ln(EC_1:5_ + 1) was strongly associated with the non-emerged position (OR = 235.0, 95% CI 10.9–5073.5, *p* < 0.001), while clay and silt were not independently significant (Table 2). When Na^+^ was added, the EC_1:5_ term remained significant (OR = 41.3, 95% CI 2.05–831.3, *p* = 0.015), whereas the Na^+^ term was not significant at the 0.05 level (OR = 7.57, 95% CI 0.72–79.6, *p* = 0.092). Replacing Na^+^ with Cl^−^ produced a similar pattern: EC_1:5_ remained significant (OR = 47.2, 95% CI 2.40–926.3, *p* = 0.011), while Cl^−^ was not independently significant at α = 0.05 (OR = 5.34, 95% CI 0.80–35.5, *p* = 0.083).

Collinearity diagnostics based on within-pair predictor differences were low for the fitted models: variance inflation factors (VIFs) ranged from 1.02–1.10 in M1, 1.09–1.56 in M2, and 1.07–1.41 in M3. Thus, although EC_1:5_ and individual ions were correlated in the broader chemical dataset, severe collinearity among the predictors included together in M1–M3 was not indicated by these pair-difference VIFs. The Na^+^ × region interaction was not significant (*p* = 0.870) after EC_1:5_ and texture were included, providing no evidence that the adjusted Na^+^ association differed statistically between Kashgar and Aksu. The very large ORs and wide confidence intervals for EC_1:5_ in the conventional conditional-logistic models indicated separation-related coefficient instability, especially in Aksu. A Firth-type bias-reduced sensitivity analysis applied to M1–M3 produced finite, less extreme estimates while preserving the main coefficient pattern: the penalized OR for standardized ln(EC_1:5_ + 1) was 86.57 in the EC + texture model; in the EC + Na + texture model, penalized ORs were 14.70 for EC_1:5_ and 5.69 for Na^+^; and in the EC + Cl + texture model, they were 18.49 for EC_1:5_ and 4.00 for Cl^−^. Thus, the direction and relative pattern of the chemical coefficients were robust to separation-reducing shrinkage, although exact OR magnitudes remain model-dependent. Overall, EC_1:5_ showed the most stable independent association with B-class status, with Na^+^ and Cl^−^ providing complementary ionic information within the broader salinity gradient.

### 2.3. Regional Ionic Signatures from PCA and Stratified Contrasts

For clarity, the PCA was used as a descriptive summary of multivariate soil chemistry (Figure 2): PC1 can be read primarily as an overall ion-concentration (salinity) axis, whereas PC2 describes a secondary contrast in ionic composition—whether the salt assemblage is relatively more Ca^2+^–SO_4_^2−^-rich or Na^+^–Cl^−^-rich. PCA of the seven major soluble ions showed that PC1 explained 54.3% of total variance and had strong positive loadings for Cl^−^ (0.865), Na^+^ (0.860), SO_4_^2−^ (0.819), Mg^2+^ (0.818), K^+^ (0.712), and Ca^2+^ (0.643), indicating a common salinity-concentration axis. PC2 explained a further 17.0% and contrasted positive Ca^2+^ (0.709) and SO_4_^2−^ (0.494) loadings with negative Na^+^ (−0.319), Cl^−^ (−0.260), K^+^ (−0.292), and HCO_3_^−^ (−0.438) loadings, thereby separating Ca^2+^–SO_4_^2−^-rich from Na^+^–Cl^−^-rich salt assemblages. The PCA is therefore used to characterize the soil-chemistry context associated with emergence status and region, not as a direct physiological test of seed germination.

The regional centroids moved in different directions along PC2 as salinity increased from A to B. In Kashgar, the centroid shifted from PC2 = +0.44 at emerged microsites to −0.41 at non-emerged microsites, whereas Aksu shifted from −0.76 to +0.71. Direct pair-level inference confirmed that these opposite shifts were not solely a visual pattern: mean ΔPC2 (B−A) was −0.850 in Kashgar and +1.472 in Aksu, giving an Aksu-minus-Kashgar difference of 2.322 (pair-bootstrap 95% CI 1.891–2.751; 9999-permutation *p* < 0.001). Regional enrichment patterns were also supported by pair-bootstrap uncertainty intervals. In Kashgar, Na^+^ enrichment was 6.22-fold (95% CI 5.08–7.66) and Cl^−^ enrichment was 5.12-fold (4.22–6.22); in Aksu, Ca^2+^ enrichment was 4.80-fold (3.36–7.51) and SO_4_^2−^ enrichment was 4.56-fold (3.58–6.03) (Figure 3). The Na^+^/(Ca^2+^ + Mg^2+^) mass ratio increased from 0.51 to 1.23 in Kashgar but decreased from 0.81 to 0.41 in Aksu.

A complementary centered-log-ratio (clr) analysis formally evaluated relative ionic composition independently of overall concentration scale. PCA of the seven-ion clr coordinates yielded a first component explaining 41.2% of compositional variance, with positive loadings for Ca^2+^ (+0.637) and SO_4_^2−^ (+0.389) and negative loadings for Na^+^ (−0.517) and Cl^−^ (−0.382). The mean paired clr-PC1 shift was −1.005 in Kashgar and +1.096 in Aksu. More importantly, the complete seven-dimensional within-pair clr-change vectors differed significantly between regions (pseudo-F = 42.49, R^2^ = 0.249, 9999-permutation *p* < 0.001), providing direct multivariate support for regional ionic-composition divergence.

### 2.4. Paired Shifts in Salinity, Salt Type, and pH Classes

The categorical comparison was intended to answer a simple paired question: did adjacent emerged (A) and non-emerged (B) microsites fall into different predefined classes of salinity severity, dominant salt type, or pH? Salinity severity refers to classes based on total water-soluble salt concentration; salt chemical type refers to classes based on the Cl^−^/SO_4_^2−^ mass ratio; and pH classes were <8.0, 8.0–8.5, and >8.5. Because A and B were paired within fields, marginal class distributions were compared with Stuart–Maxwell tests rather than independent-sample χ^2^ tests (Table 3). Salinity severity changed strongly from A to B (Stuart–Maxwell χ^2^ = 84.50, df = 4, *p* < 0.001). At A positions, 30.8% of samples were non-saline and 30.0% slightly saline, whereas no B sample was non-saline; 51.5% of B samples were severely saline and 14.6% were extremely saline (>20 g kg^−1^). Salt chemical type also shifted significantly (χ^2^ = 6.83, df = 2, *p* = 0.033), with sulfate–chloride samples increasing from 13.8% at A to 25.4% at B. pH class changed as well (χ^2^ = 10.57, df = 2, *p* = 0.005), largely because high-pH samples (pH > 8.5) were more frequent at B positions. Regionally, however, pH increased from A to B in Kashgar (8.25 to 8.53) but decreased in Aksu (8.43 to 8.23), so elevated pH was not a universal feature of emergence failure.

### 2.5. Pair-Bootstrap Diagnostic Performance

Pair-bootstrap ROC analysis showed that no single gravimetric total-salt cut-point was transferable between regions (Table 4; Figure 4). In the combined dataset, Na^+^ had the highest AUC (0.909; 95% CI 0.875–0.942), with a Youden cut-point of 0.546 g kg^−1^ (bootstrap 95% CI 0.422–0.728), sensitivity 0.80, and specificity 0.915. EC_1:5_ had a similar AUC of 0.902 (95% CI 0.877–0.928) and a combined cut-point of 2.23 mS cm^−1^. Cl^−^ also discriminated well (AUC 0.892).

Regional apparent performance differed markedly. In Kashgar, Cl^−^ (AUC 0.953), Na^+^ (0.944), and SAR_1:5_ (0.902) were strong discriminators; EC_1:5_ had an AUC of 0.898 with a cut-point of 2.51 mS cm^−1^, and total salt had an AUC of 0.842 with a cut-point of 13.7 g kg^−1^. In Aksu, total salt and EC_1:5_ both showed near-perfect apparent discrimination (AUC 0.999), with cut-points of 5.0 g kg^−1^ and 1.17 mS cm^−1^, respectively, whereas SAR_1:5_ performed poorly (AUC 0.597). Given the smaller Aksu sample (40 pairs) and deliberate selection of strongly contrasting A/B microsites, an AUC of 0.999 is also consistent with small-sample separation and should not be interpreted as a clean or transportable regional diagnostic signal. These values describe discrimination within the deliberately contrast-selected sampled spectrum and should not be interpreted as expected performance across unselected commercial-field conditions.

Pair-preserving leave-one-pair-out (LOPO) cross-validation produced only modest changes in EC_1:5_ classification within the sampled contrast. In Kashgar, EC_1:5_ sensitivity/specificity changed from apparent values of 0.722/0.967 to LOPO values of 0.711/0.967 (balanced accuracy 0.839). In Aksu, the corresponding values changed from 1.000/0.975 to 0.950/0.975 (balanced accuracy 0.963). For total salt, LOPO sensitivity/specificity were 0.556/0.956 in Kashgar and 0.950/1.000 in Aksu. The smaller Aksu sample (40 pairs versus 90 in Kashgar) and deliberate selection of strongly contrasting A/B microsites warrant particular caution despite the high internally validated classification values. These cut-points remain exploratory and require external validation.

## 3. Discussion

### 3.1. A Common Seed-Zone Salinity Gradient Is the Dominant Association

At the survey date, the most consistent pattern across the 130 commercial fields was higher measured seed-zone salinity at non-emerged microsites than at adjacent emerged microsites within the same field. The paired design is important because A and B positions shared many field-level management conditions, including cultivar, sowing date, planting density, mulch and drip layout, fertilization, and tillage history. However, soil sampling occurred 14–28 d after sowing, after emergence status had become apparent. Consequently, the study cannot establish that the measured salinity differences preceded non-emergence. Plant water use, evaporation, irrigation redistribution, and other post-emergence soil–water dynamics may also have contributed to the measured A–B contrasts. The findings should therefore be interpreted as cross-sectional within-field associations at the survey date rather than proof that the measured salinity caused the preceding failure to emerge. Within that limitation, the paired results extend previous experimental evidence on emergence-period water and salt conditions in southern Xinjiang [11,12,13] by demonstrating agronomically relevant salinity contrasts within conventionally managed commercial fields. Because soil water content, soil temperature, matric potential, and osmotic potential were not measured directly, the survey also cannot distinguish salinity acting alone from salinity interacting with transient water limitation or thermal conditions during germination and emergence.

A particularly important competing explanation is post-outcome evaporative concentration at non-emerged microsites. Because B locations lacked an established stand, the seed-hole and near-surface zone may have remained more exposed to evaporation than at A locations; under the high evaporative demand of southern Xinjiang, post-irrigation evaporation could concentrate salts in the sampled 0–10 cm layer after the emergence outcome was already established. The observed B > A salinity contrast may therefore reflect a combination of antecedent salinity stress and consequence-related evaporative concentration, and the present cross-sectional sampling cannot partition these components. A future causal design should obtain pre-sowing or pre-emergence baseline samples at the same paired locations and repeat measurements through irrigation, germination, and early establishment, while recording soil water content or soil-solution EC, matric and osmotic potentials, emitter discharge, microtopography, and local evaporative or canopy conditions. Such longitudinal measurements would allow salinity changes that precede emergence failure to be distinguished from changes that develop after failed establishment.

The matched multivariable models helped clarify the univariate ion associations. Na^+^ showed the greatest fold enrichment and the highest AUC in the combined dataset, but it was strongly correlated with EC_1:5_ and total salt. Pair-difference VIFs were nevertheless low within the fitted models (maximum 1.56), indicating that the instability of the very large conventional EC_1:5_ odds ratios is better attributed to outcome separation than to severe multicollinearity among predictors entered together. After adjustment for EC_1:5_ and soil texture, EC_1:5_ remained significantly associated with non-emergence, whereas the Na^+^ term was no longer significant at the 0.05 level (*p* = 0.092); a similar pattern was observed for Cl^−^. These results indicate that the overall salinity gradient, represented by EC_1:5_, showed the most stable field-level association with non-emergence, whereas Na^+^ and Cl^−^ primarily reflected differences in the ionic composition of that gradient. Given the broader correlation structure among chemical variables, the large univariate associations of individual ions should not be interpreted as evidence that any single ion independently caused non-emergence.

This interpretation is consistent with established plant physiological responses to salinity. High external salinity reduces water uptake and can delay or inhibit germination, while elevated Na^+^ and Cl^−^ may impose additional ion-specific stress after imbibition and root emergence [14,15,16,17]. The present field-based observational design cannot experimentally separate osmotic effects from ion-specific effects, but it can identify the soil indicators that most effectively distinguish seed-zone microsites associated with non-emergence.

### 3.2. Regional Ionic Signatures: Na^+^–Cl^−^ in Kashgar and Ca^2+^–SO_4_^2−^ in Aksu

The regional ionic-composition result is supported by both the paired change in the original PCA composition-related axis and a clr-based multivariate analysis that removes overall concentration scale from the composition coordinates. Mean ΔPC2 was −0.850 in Kashgar and +1.472 in Aksu, with a significant between-region difference (2.322; 95% CI 1.891–2.751; *p* < 0.001). The full seven-ion clr-change vectors also differed by region (pseudo-F = 42.49, R^2^ = 0.249, *p* < 0.001). Kashgar pairs therefore shifted toward relatively greater Na^+^–Cl^−^ representation, whereas Aksu pairs shifted toward relatively greater Ca^2+^–SO_4_^2−^ representation. These direct inferential results support describing the observed patterns as regional ionic signatures, while the observational design does not establish that different ions were independently causal in the two regions.

Regional differences in salt chemistry may arise from variation in irrigation-water composition, parent material, groundwater influence, evaporative concentration, and mineral precipitation–dissolution processes. Sulfate-rich and chloride-rich salt assemblages differ in solubility and mobility, and similar EC values can therefore reflect different ionic compositions. Because irrigation-water chemistry and mineral-phase composition were not systematically measured in this survey, the processes underlying the observed regional patterns cannot be determined from the present data. Nevertheless, the PCA results and regional ion-enrichment patterns indicate that salinity assessment in southern Xinjiang should account for both overall salinity and ionic composition rather than assuming a spatially uniform NaCl-dominated system.

The Na^+^ × region interaction was not significant after adjustment for EC_1:5_ and soil texture (*p* = 0.870), providing no evidence that the adjusted association between Na^+^ and emergence status differed between Kashgar and Aksu. This interaction and the compositional tests address different questions: the interaction evaluates effect modification of the adjusted Na^+^ association, whereas the paired PC2 and clr analyses test whether the multivariate ionic composition of the A–B change differs by region. The significant compositional tests, together with the non-significant Na^+^ interaction, support a common salinity-related association with non-emergence accompanied by regionally distinct ion assemblages, without implying region-specific causation by a single ion.

### 3.3. Soil Texture as Physical Context Rather than Evidence of Sodium-Induced Structural Degradation

Non-emerged microsites had consistently coarser particle-size distributions, characterized primarily by lower silt and higher fine-sand contents. However, this pattern should not be interpreted as direct evidence of sodium-induced clay dispersion. Particle-size analysis following chemical dispersion quantifies mineral particle-size fractions rather than aggregate stability, and the observed reduction in clay content cannot reasonably be attributed to dispersion occurring over the short emergence period. The differences in particle-size distribution are therefore more consistent with pre-existing within-field textural heterogeneity that may interact with water movement and salt redistribution.

Soil texture influences pore-size distribution, infiltration, water retention, and evaporation, all of which can affect salt accumulation around shallowly planted cotton seeds. Coarser microsites may drain or dry more rapidly following emergence irrigation, potentially increasing salt concentrations in the upper seed zone, whereas finer-textured microsites may retain water for longer periods. Conversely, elevated exchangeable sodium can impair soil structure and hydraulic conductivity under sufficiently sodic conditions [18,21,22], but such effects cannot be inferred from SAR_1:5_ and particle-size fractions alone. Direct measurements of exchangeable sodium percentage (ESP), cation exchange capacity (CEC), aggregate stability, bulk density, penetration resistance, and hydraulic conductivity would be required to distinguish inherent textural effects from sodium-induced structural degradation.

### 3.4. Exploratory Field Diagnostic Cut-Points and Management Implications

The pair-bootstrap ROC analysis provides a practical framework for exploratory field screening while also illustrating why a single diagnostic threshold is unlikely to be appropriate across southern Xinjiang. In Aksu, EC_1:5_ and total salt showed near-perfect apparent discrimination at cut-points of approximately 1.17 mS cm^−1^ and 5.0 g kg^−1^, respectively; in Kashgar, the corresponding cut-points were higher, at 2.51 mS cm^−1^ for EC_1:5_ and 13.7 g kg^−1^ for total salt. Pair-preserving LOPO validation caused only modest changes in EC_1:5_ classification, with balanced accuracy of 0.839 in Kashgar and 0.963 in Aksu. Nevertheless, Aksu contributed only 40 pairs compared with 90 in Kashgar, and the survey deliberately selected strongly contrasting emerged and non-emerged microsites. The Aksu AUC of 0.999 is therefore especially compatible with small-sample separation under strong contrast selection and should not be interpreted as a clean regional diagnostic signal. These internally derived cut-points should be regarded as screening values for field conditions similar to those sampled here, not as externally validated thresholds, physiological salt-tolerance limits, or estimates of emergence probability; independent external validation is required before field use.

EC_1:5_ may therefore serve as a useful first-line field indicator because it remained significantly associated with non-emergence in the matched multivariable models and can be measured rapidly using portable instruments. Na^+^ and Cl^−^ may provide complementary information, particularly in Kashgar, where their AUC values exceeded that of EC_1:5_. These findings support a two-stage field assessment approach: first identify seed-zone areas with elevated EC_1:5_, and then characterize ionic composition where recurrent emergence failure occurs. Such information could help target subsequent irrigation or leaching trials to affected areas rather than applying uniform interventions across entire fields. Previous experiments in southern Xinjiang have shown that adjusting the timing and frequency of emergence irrigation can improve seed-zone water and salt conditions and promote cotton establishment [12,13].

Recommendations for chemical amendments should remain conditional on additional soil measurements. Gypsum may be appropriate where exchangeable sodium is demonstrably elevated and drainage is adequate, but soluble Na^+^ concentrations or SAR_1:5_ measured in a 1:5 extract are insufficient on their own to determine gypsum requirements. Measurements of exchangeable sodium percentage (ESP), cation exchange capacity (CEC), soil carbonate status, irrigation-water quality, and drainage capacity are needed before amendment decisions are made [23,24,25]. Similarly, the EC_1:5_ values reported here should not be directly substituted into crop salt-tolerance functions based on saturated-paste ECe because the two extraction methods differ in soil-to-water ratio and ionic composition.

### 3.5. Limitations and Priorities for Validation

Several limitations constrain the causal and diagnostic interpretation of these findings. First, this was a cross-sectional paired observational survey conducted during a single cotton establishment period, and soil sampling occurred after field-establishment status had already become apparent. The measured salinity therefore cannot be shown to have preceded non-emergence, and reverse or bidirectional influences through plant water use, evaporation, irrigation redistribution, and other post-emergence processes cannot be excluded. Soil water content, soil temperature, matric potential, osmotic potential, microtopography, emitter discharge uniformity, and actual plot-scale irrigation amounts were not measured consistently enough to be included in the statistical models. Mechanized sowing in the surveyed fields typically placed cotton seeds at approximately 1.0–1.5 cm depth, but exact seed placement depth was not measured at every sampled seed hole. Consequently, the present data cannot determine whether poor field establishment was associated with salinity acting alone, with water or temperature stress, or with interactions among these factors. In addition, EC_1:5_ is a standardized laboratory extract for comparative soil characterization and is not a direct measurement of in situ soil-solution salinity, osmotic potential, or seed-zone water potential. The composite 0–10 cm samples integrate the shallow soil environment that contains the sowing depth but average vertical heterogeneity and therefore do not reproduce the exact microscale soil-solution conditions at the seed–soil interface. Residual heterogeneity in irrigation amount, timing, emitter performance, microtopography, texture, and water redistribution may therefore confound part of the observed A–B association despite within-field pairing.

Second, the sampling design intentionally maximized within-field contrast. Fields were included for paired sampling only when a relatively large area with no seedlings or only very sparse establishment was clearly identifiable. Within such fields, B microsites were selected in the poor-establishment patch and defined operationally as having <5% emergence, whereas A microsites were selected within the surrounding field area where the live-seedling stand was ≥80%. This non-random, visually guided contrast selection is appropriate for detecting soil-property differences between paired microsites but can introduce selection subjectivity and spectrum bias and can inflate apparent diagnostic discrimination relative to unselected field conditions. It does not estimate the prevalence of emergence failure or the absolute probability of establishment at a given salinity level. Beyond the operational <5% and ≥80% classification thresholds, exact continuous emergence or stand percentages were not recorded consistently across all fields; therefore, the binary response cannot characterize a continuous dose–response relationship. Future validation should use probabilistic sampling of seed positions and record emergence percentage or plant density quantitatively. The original cross-sectional survey did not include a standardized photographic archive or a blinded independent reclassification of all microsites; the field classification therefore relied on investigator screening followed by confirmation against the predefined <5% and ≥80% operational stand criteria.

Third, mechanistic interpretation of sodium-related effects is limited by the absence of measurements of exchangeable sodium percentage (ESP), cation exchange capacity (CEC), aggregate stability, hydraulic conductivity, bulk density, and irrigation-water chemistry. SAR_1:5_ provides an internally consistent relative sodium-hazard descriptor within this dataset, but it is not equivalent to saturated-paste SAR, ESP, or direct evidence of soil sodicity. Finally, the diagnostic cut-points were derived from the same single-year, deliberately contrast-selected dataset in which their apparent performance was evaluated. Pair-preserving LOPO analysis provides internal validation of cut-point classification but does not replace independent external validation. Validation across multiple years, cultivars, irrigation regimes, more representative field spectra, and additional oasis regions is needed before these cut-points can be considered for operational field use.

Systematic time-series field photography and spatially and temporally matched remote-sensing indices (e.g., NDVI or related vegetation/salinity indices) were not components of the original cross-sectional survey. Because the study was not designed as a longitudinal imaging experiment, such records cannot be reconstructed consistently after the fact. Future work should combine repeated ground photographs, soil water and temperature measurements, and UAV or other high-resolution remote sensing with repeated seed-zone sampling so that stand development can be related directly to spatiotemporal water–salt dynamics.

## 4. Materials and Methods

### 4.1. Study Area

The survey was conducted in two major cotton-producing oasis regions of southern Xinjiang, China: Kashgar Prefecture (71°23′–79°52′ E, 35°28′–40°16′ N) on the southwestern margin of the Taklamakan Desert and Aksu Prefecture (79°30′–82°00′ E, 40°20′–41°50′ N) on the northern margin of the Tarim Basin (Figure 5). Both regions have warm-temperate continental arid climates, high evaporative demand, and irrigation-dependent agriculture. Mean annual precipitation is approximately 35 mm in Kashgar and 50–75 mm in Aksu, whereas annual potential evaporation is approximately 2600 and 1900–2300 mm, respectively. Dominant soil groups include Calcisols and Arenosols, with irrigated alluvial soils also common in Aksu [26,27]. Drip irrigation under plastic mulch is widely used for cotton production in both regions. All observations and soil sampling in the present study were conducted in open commercial cotton fields under normal farmer management; no greenhouse, pot, or controlled-environment experiment was involved.

### 4.2. Field Survey and Paired Soil Sampling

Sampling was conducted on 10–14 May 2025 during cotton establishment, approximately 14–28 d after sowing. A total of 130 commercial cotton fields were surveyed, including 90 in Kashgar and 40 in Aksu. During field screening, the field-survey investigators (T.Q., J.H., and Z.W.) first visually inspected each field for the presence of a relatively large patch with no seedlings or only very sparse seedling establishment. Candidate microsites identified by this initial visual screen were then checked against the operational stand criteria used for sampling: B microsites had <5% emergence, whereas A microsites had a live-seedling stand ≥80%. Paired sampling was conducted only when a clearly identifiable poor-establishment patch was present. Within each selected field, a non-emerged or nearly non-emerged microsite (B) was located within this patch, and the corresponding well-established microsite (A) was selected within approximately 50–100 m of the B microsite in a nearby portion of the same field. The paired A and B microsites shared the same field-level management history, including cultivar, sowing date, planting density, plastic mulch configuration, drip-irrigation layout, fertilization, and tillage history. The response was therefore analyzed as a binary field-establishment category contrasting well-established A and non-emerged or nearly non-emerged B microsites rather than as a continuous emergence-rate response. A and B were treated as within-field paired locations rather than environmentally identical microsites; local variation in microtopography, emitter performance, soil texture, and water redistribution could remain. Because field classification involved visual selection of strongly contrasting stands, some selection subjectivity and spectrum bias are possible and are addressed in the limitations. Because soil was sampled after the A/B field-establishment classes had been identified, the measurements represent conditions observed at the survey date and do not establish temporal precedence relative to germination or emergence failure.

Cotton in the surveyed fields was established by mechanized sowing under plastic-mulched drip irrigation, with a typical sowing depth of approximately 1.0–1.5 cm. At each A and B microsite, soil was collected from the standardized 0–10 cm layer using a stainless-steel auger (3 cm internal diameter). This interval was operationally defined as the shallow seed-zone layer because it encompassed the typical sowing depth and the upper soil volume expected to exert the strongest direct influence on cotton seeds and young seedlings from sowing through early establishment. In this near-surface layer, irrigation redistribution, evaporation, and associated salt accumulation can rapidly modify the local water–salt environment. Each composite sample consisted of five subsamples collected from adjacent seed holes within the same microsite to reduce hole-scale variability and provide an integrated characterization of the local shallow seed-zone environment. Because the samples represented a composite 0–10 cm interval, they inherently averaged vertical heterogeneity and therefore should not be interpreted as reproducing the exact soil-solution conditions immediately adjacent to individual seeds during germination. Samples were air-dried in the shade, gently crushed, passed through a 2-mm nylon sieve, and stored in sealed polyethylene bags until analysis. The final dataset comprised 260 soil samples forming 130 complete A–B field pairs, including 90 pairs from Kashgar and 40 pairs from Aksu. Field records included geographic coordinates, cultivar, sowing date, field-establishment status, salt-damage observations, mulching and drip-irrigation configuration, and farmer-reported irrigation and fertilization information. Irrigation amount and frequency were not included as model covariates because farmer-reported records were insufficiently complete and precise across all surveyed fields.

### 4.3. Laboratory Analyses

Soil analyses were performed at the Institute of Agricultural Resources and Environment Testing Laboratory, Xinjiang Academy of Agricultural Sciences, according to standard soil analytical methods described by Bao [28]. Soil pH was measured in a 1:2.5 soil-to-water suspension. Electrical conductivity was measured in a 1:5 soil-to-water extract and is reported throughout as EC_1:5_ (mS cm^−1^); no conversion to saturated-paste ECe was used in the inferential analyses or diagnostic cut-points. EC_1:5_ was used as a standardized comparative soil-characterization measure and should not be interpreted as a direct measurement of in situ soil-solution salinity, osmotic potential, or seed-zone water potential. Total water-soluble salts were determined gravimetrically after evaporation of a filtered 1:5 extract.

Major soluble ions in the 1:5 extract were determined as follows: CO_3_^2−^ and HCO_3_^−^ by double-indicator titration with H_2_SO_4_; Cl^−^ by AgNO_3_ titration (Mohr method); SO_4_^2−^ by EDTA indirect titration after BaCl_2_ precipitation; Ca^2+^ and Mg^2+^ by EDTA complexometric titration; and Na^+^ and K^+^ by flame photometry. Ion concentrations are reported on an oven-dry soil mass basis (g kg^−1^). CO_3_^2−^ was below detection in all samples. Agreement between the summed measured ions and gravimetric total salt was high (r^2^ = 0.98; mean recovery 92.0% ± 11.3%, *n* = 260), supporting analytical consistency.

Particle-size distribution was measured by the Bouyoucos hydrometer method after dispersion with sodium hexametaphosphate. Fractions were clay (<0.002 mm), silt (0.002–0.02 mm), fine sand (0.02–0.2 mm), and coarse sand (0.2–2 mm), consistent with the Chinese classification framework [29].

### 4.4. Derived Indices and Categorical Classifications

The sodium adsorption ratio from the 1:5 extract (SAR_1:5_) was calculated from charge-equivalent cation concentrations and is reported explicitly as an extract-specific relative sodium-hazard index rather than as saturated-paste SAR. Concentrations were converted from g kg^−1^ soil to meq L^−1^ extract with the 1:5 dilution accounted for: Ca^2+^ = Ca^2+^ (g kg^−1^) × 1000/20.04/5; Mg^2+^ = Mg^2+^ (g kg^−1^) × 1000/12.15/5; Na^+^ = Na^+^ (g kg^−1^) × 1000/22.99/5. SAR_1:5_ was then calculated as Na^+^/√[(Ca^2+^ + Mg^2+^)/2] [30]. The Na^+^/(Ca^2+^ + Mg^2+^) mass ratio was retained as a complementary descriptor of sodium dominance, not as a substitute for ESP or standard sodicity indices.

For clarity, the categorical analyses used three separate descriptors rather than a single combined classification. Salt chemical type was classified from the Cl^−^/SO_4_^2−^ mass ratio as sulfate (<0.5), sulfate–chloride (0.5–1.0), chloride–sulfate (1.0–4.0), or chloride (>4.0). Salinity severity was classified independently from total water-soluble salts as non-saline (<3 g kg^−1^), slightly saline (3–6 g kg^−1^), moderately saline (6–10 g kg^−1^), severely saline (10–20 g kg^−1^), or extremely saline (>20 g kg^−1^) following Chinese soil survey conventions [31]. For the paired pH-class analysis, pH was grouped as <8.0, 8.0–8.5, and >8.5; the >8.5 category is referred to in the text as high-pH samples. These classifications were used only to summarize paired shifts in field-condition categories and were not treated as mechanistic thresholds for cotton emergence.

### 4.5. Statistical Analysis

All inferential analyses accounted for the within-field paired design. Paired differences between well-established A microsites (live-seedling stand ≥ 80%) and non-emerged or nearly non-emerged B microsites (emergence < 5%) were evaluated using Wilcoxon signed-rank tests because salinity and ion variables were right-skewed and many paired-difference distributions deviated from normality. The Benjamini–Hochberg procedure was applied to control the false discovery rate across the 17 paired tests at q = 0.05. Hodges–Lehmann estimators were used to summarize robust paired effect sizes, and the proportion of field pairs in which B exceeded A was reported for key indicators.

Because A and B samples were matched within fields, distributions of salinity class, salt type, and pH class were compared using Stuart–Maxwell tests of marginal homogeneity rather than Pearson χ^2^ tests for independent samples. Pearson correlation coefficients were used descriptively to assess collinearity among continuous variables. Total salt and EC_1:5_ were not included simultaneously in multivariable models because they were strongly correlated (r = 0.982).

Matched conditional logistic regression was used to identify soil properties independently associated with the non-emerged microsite, with each field pair treated as a stratum. Positive-valued chemical variables were transformed as ln(x + 1) and subsequently standardized to a mean of 0 and an SD of 1; soil texture fractions were standardized without transformation. A prespecified base model included EC_1:5_, clay, and silt. Clay and silt were selected as the finer texture fractions relevant to water retention and seed-zone hydraulic behavior; because the four particle-size fractions form a constrained composition that sums to approximately 100%, fine and coarse sand were omitted to avoid exact or near-exact redundancy. Texture terms were used as covariate adjustment rather than interpreted as independent mechanistic effects. A second model additionally included Na^+^, whereas a third model included Cl^−^ in place of Na^+^. A Na^+^ × region interaction term was subsequently included to test whether the adjusted association between Na^+^ and emergence status differed between Kashgar and Aksu after accounting for EC_1:5_ and soil texture. An analogous EC_1:5_ × region interaction model was explored but exhibited quasi-complete separation because EC_1:5_ almost perfectly discriminated emerged from non-emerged microsites in Aksu; the unstable coefficient estimates were not used for inference. Odds ratios (ORs) and 95% confidence intervals (CIs) are reported per 1-SD increase in transformed and standardized predictors. To assess collinearity in a manner aligned with the matched-pair information, variance inflation factors (VIFs) were calculated from the pair-level predictor differences (xB − xA) using the same transformed predictor definitions as M1–M3; scaling does not affect VIF. Because the conventional conditional-logistic models showed large coefficients and wide confidence intervals, a Firth-type bias-reduced sensitivity analysis was additionally applied to M1–M3 using the matched-pair conditional likelihood expressed through within-pair predictor differences. This penalized analysis was used as a separation-robust sensitivity check of coefficient direction and relative magnitude, while the conventional conditional-logistic models remained the primary inferential analysis.

To characterize covariance in ion concentrations, HCO_3_^−^, Cl^−^, SO_4_^2−^, Ca^2+^, Mg^2+^, K^+^, and Na^+^ were transformed as ln(x + 1), standardized, and subjected to principal component analysis (PCA). For interpretation, PC1 was treated primarily as an overall ion-concentration/salinity axis, whereas PC2 was treated as a secondary composition-related axis contrasting Ca^2+^–SO_4_^2−^-rich with Na^+^–Cl^−^-rich assemblages. Region- and emergence-status-specific PCA centroids were used to describe these two multivariate soil-chemistry patterns; the PCA components were not interpreted as direct germination-response variables. To directly test whether the paired change along PC2 differed between regions, ΔPC2 was calculated for each field pair as PC2B − PC2A. Regional mean ΔPC2 values were compared using a pair-level permutation test (9999 label permutations), and the between-region difference was summarized with a pair-resampling bootstrap 95% CI. All 260 soil samples were retained in the primary PCA, and no observations were excluded solely because of extreme positions in PCA score space.

As a complementary analysis of relative ionic composition, the seven positive ion concentrations were transformed to centered-log-ratio (clr) coordinates, clr(xij) = ln(xij) − meanj[ln(xij)]. All measured concentrations were positive, so no zero replacement was required. PCA was applied to the clr coordinates without variable standardization. For each field pair, the seven-dimensional clr change vector was calculated as clr(B) − clr(A). Regional differentiation of this paired compositional change was evaluated with a Euclidean-distance permutation pseudo-F statistic in clr space using 9999 permutations of region labels. R^2^ was calculated as the proportion of total pair-level clr-change sum of squares attributable to region.

Receiver operating characteristic (ROC) analysis was used to evaluate field discrimination rather than to estimate physiological salt-tolerance thresholds. To preserve within-field dependence, uncertainty was quantified by resampling complete A–B field pairs with replacement in 1000 bootstrap replicates for the combined dataset and separately for Kashgar and Aksu. For each replicate, the area under the ROC curve (AUC) and the cut-point maximizing Youden’s J statistic were recalculated, and percentile bootstrap distributions were used for 95% CIs. Because bootstrap uncertainty does not constitute independent validation, cut-point classification was additionally evaluated using pair-preserving leave-one-pair-out (LOPO) cross-validation. In each iteration, one complete A–B pair was held out, the Youden cut-point was estimated from the remaining pairs, and that threshold was applied to both observations in the held-out pair; pooled held-out predictions were used to calculate sensitivity, specificity, and balanced accuracy. LOPO validation was conducted for the combined dataset and separately within Kashgar and Aksu for Na^+^, EC_1:5_, Cl^−^, total salt, and SAR_1:5_. Because the sampling design deliberately contrasted successfully emerged and non-emerged microsites, both apparent and cross-validated results remain internal to the sampled spectrum and should not be interpreted as externally validated emergence probabilities.

For Figure 3, regional B/A ion-enrichment ratios were calculated as the ratio of the regional mean concentration at B microsites to that at A microsites. Ninety-five percent confidence intervals were obtained by resampling complete field pairs with replacement within each region (10,000 replicates). The observed ratios and confidence limits were transformed to log_2_(B/A) for plotting.

Data management and statistical analyses were performed using Microsoft Excel 2019, OriginPro 2024, and Python 3.12 with pandas 2.2, NumPy 1.26, SciPy 1.13, statsmodels 0.14, and Matplotlib 3.8. All statistical results reported in the manuscript were recalculated from the original datasets and checked by the authors before manuscript finalization.

## 5. Conclusions

Across 130 commercial cotton fields, non-emerged or nearly non-emerged B microsites (<5% emergence) had consistently higher measured soluble-salt concentrations than paired well-established A microsites (live-seedling stand ≥ 80%) at the survey date under shared field-level management. Because soil sampling occurred after field-establishment status had become apparent, these results demonstrate a strong cross-sectional association but do not establish that the measured salinity preceded or caused the earlier emergence failure; post-outcome evaporative concentration remains a plausible competing explanation for part of the B-site salinity enrichment. The standardized 0–10 cm samples should be interpreted as an integrated shallow seed-zone characterization that encompasses the typical 1.0–1.5 cm sowing depth, rather than as a direct measurement of microscale soil-solution conditions at the seed–soil interface. Matched multivariable models showed that EC_1:5_ retained the most stable independent association with B-class status after adjustment for soil texture and either Na^+^ or Cl^−^, and low pair-difference VIFs together with separation-robust sensitivity analysis supported the qualitative coefficient pattern. Direct pair-level PC2 inference and clr-based multivariate analysis supported contrasting regional ionic compositions: Kashgar pairs shifted toward relatively greater Na^+^–Cl^−^ representation, whereas Aksu pairs shifted toward relatively greater Ca^2+^–SO_4_^2−^ representation. Pair-bootstrap ROC analysis yielded region-specific EC_1:5_ field diagnostic cut-points of approximately 2.51 mS cm^−1^ in Kashgar and 1.17 mS cm^−1^ in Aksu, and pair-preserving LOPO analysis provided internal validation of cut-point classification. These values remain exploratory and internally derived from a deliberately contrast-selected single-year dataset; they are not physiological salt-tolerance thresholds or externally validated operational criteria. Independent external validation across additional fields, years, cultivars, and irrigation settings is required before these cut-points are used for field decision-making. Seed-zone EC_1:5_ screening, complemented by ion analysis where appropriate, may provide a framework for identifying areas associated with poor establishment and for guiding subsequent irrigation, leaching, and validation trials.

## Figures and Tables

**Figure 1:**
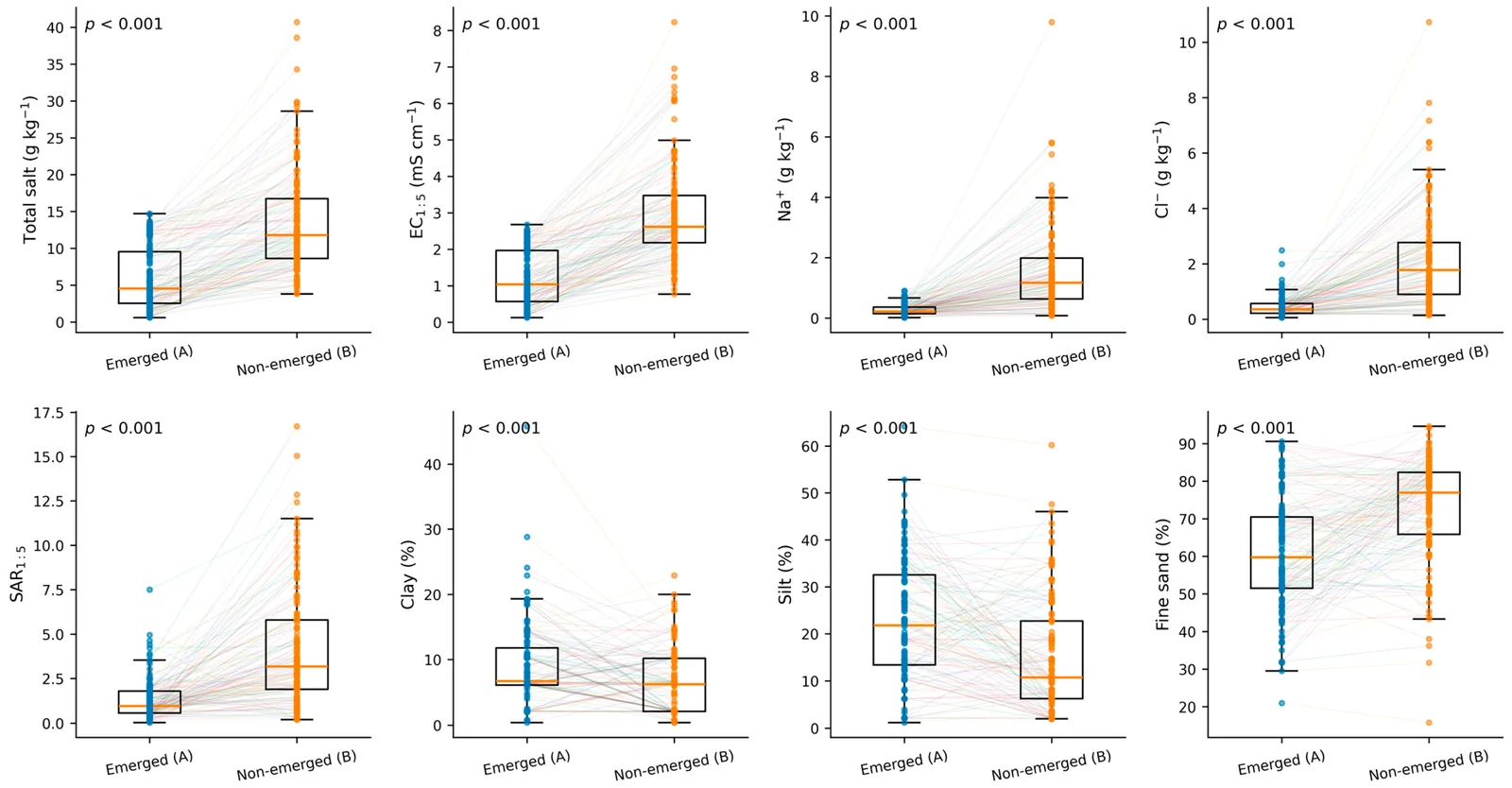
Within-field paired contrasts in key seed-zone soil properties across 130 complete A–B field pairs. Lines connect emerged (A) and non-emerged (B) microsites from the same field; *p*-values are from paired Wilcoxon signed-rank tests, and FDR correction did not change significance conclusions.

**Figure 2:**
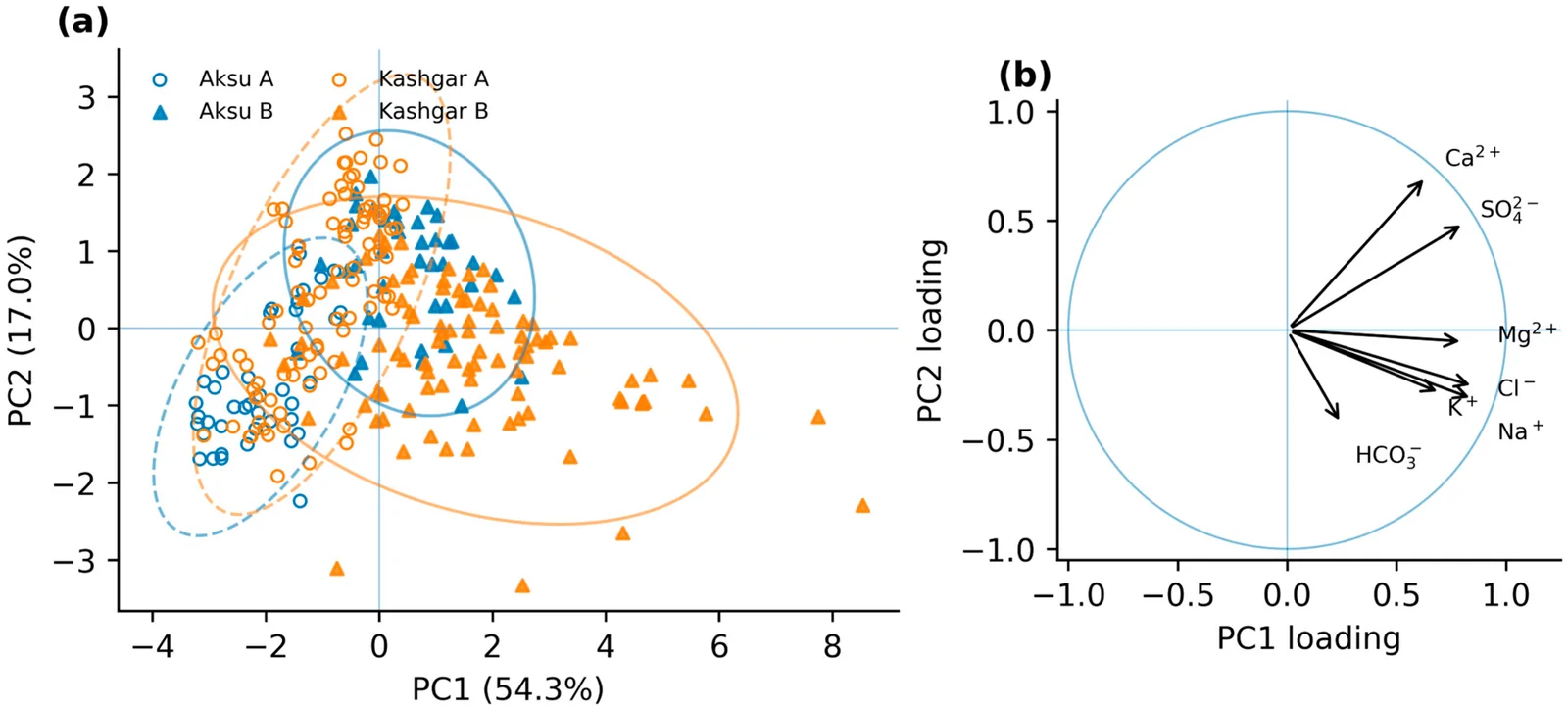
Principal component analysis of seven major soluble ions across all 260 soil samples. (**a**) PCA scores by region and emergence status; ellipses summarize group dispersion. (**b**) Correlation loadings for the seven ions. PC1 explained 54.3% and primarily summarizes overall ion concentration, whereas PC2 explained 17.0% and summarizes the secondary Ca^2+^–SO_4_^2−^ versus Na^+^–Cl^−^ composition contrast. PCA is used here as a multivariate description of soil chemistry rather than as a direct germination-response model.

**Figure 3:**
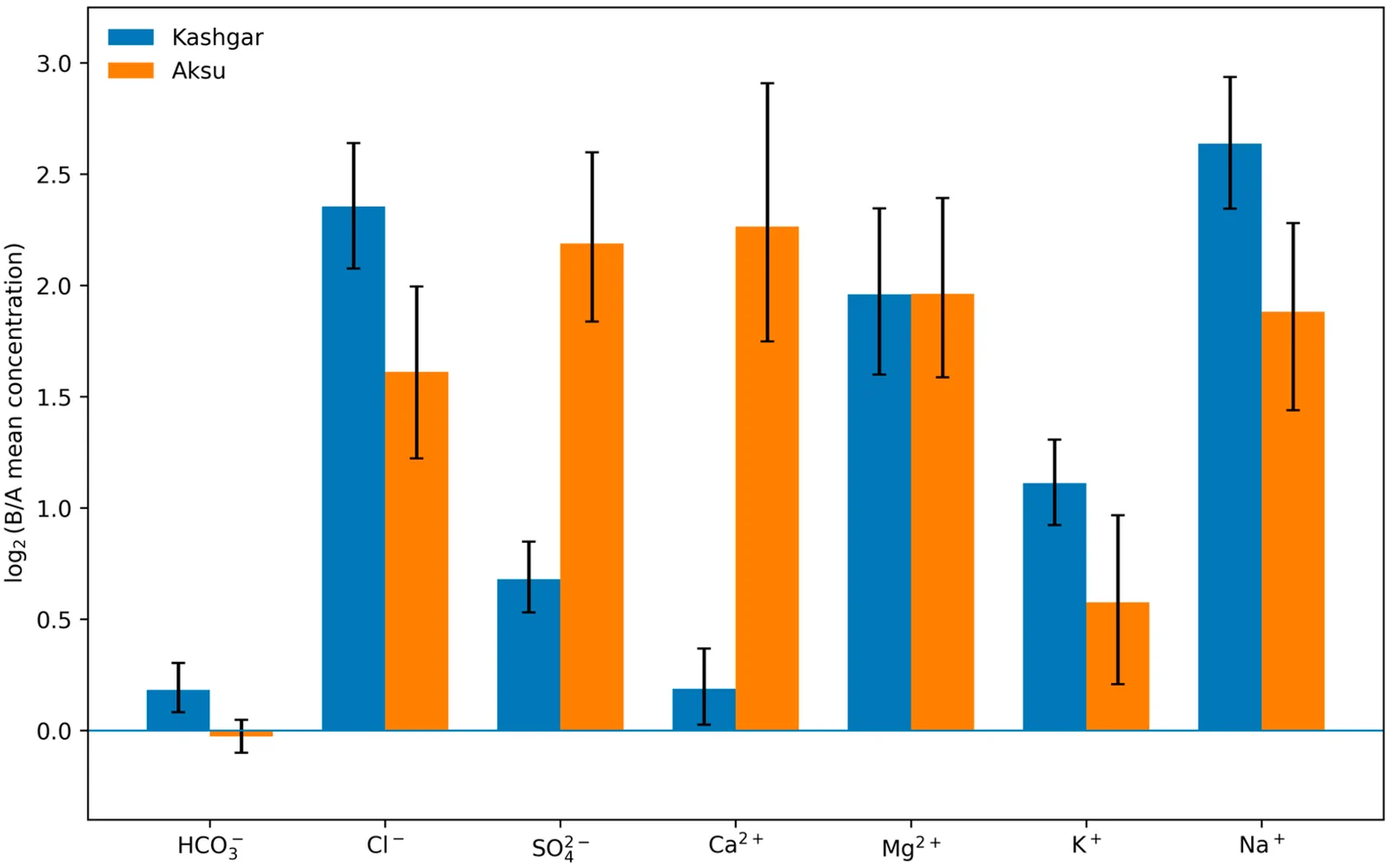
Region-specific ionic enrichment at non-emerged microsites. Bars show log_2_-transformed B/A ratios of regional mean ion concentrations; error bars are percentile 95% confidence intervals obtained by resampling complete field pairs with replacement within each region (10,000 replicates). Kashgar showed stronger Na^+^–Cl^−^ enrichment, whereas Aksu showed stronger Ca^2+^–SO_4_^2−^ enrichment.

**Figure 4:**
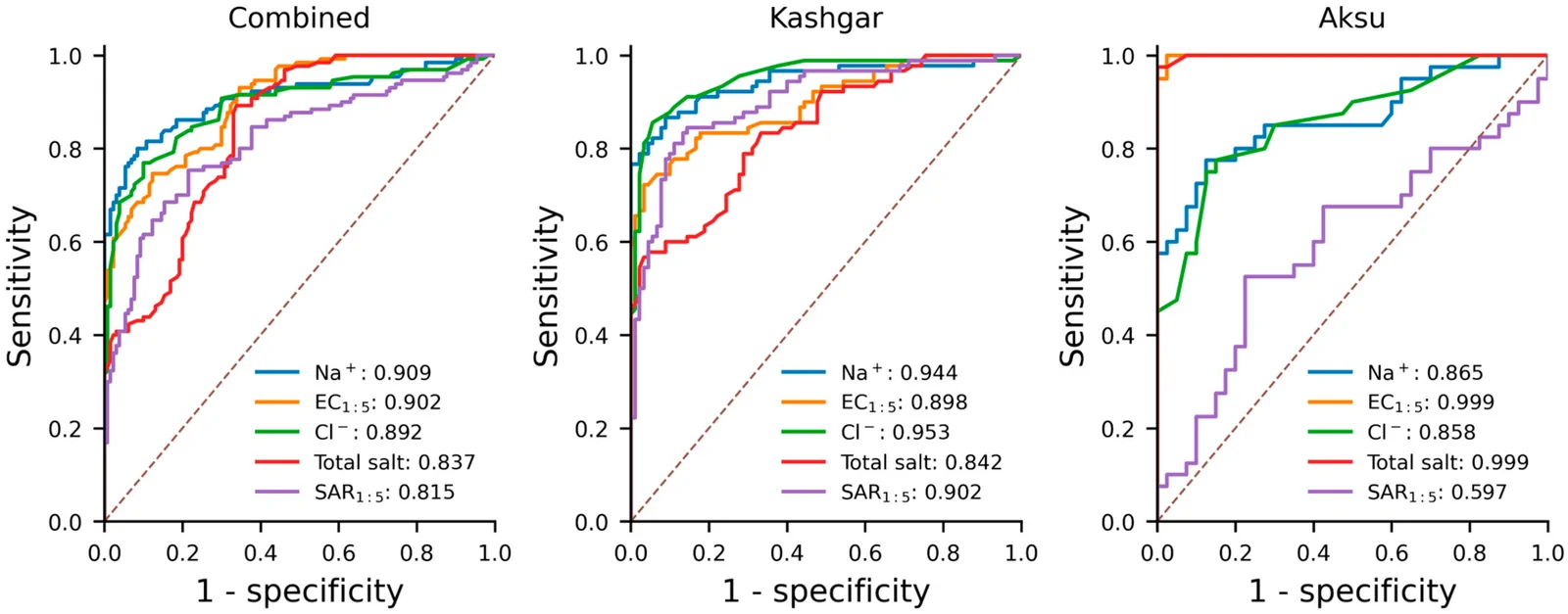
Diagnostic discrimination of key seed-zone indicators in the combined dataset and separately in Kashgar and Aksu. Curves show empirical ROC relationships in the observed samples; the red dashed diagonal line represents the random classifier (AUC = 0.5). AUC values in the legends represent apparent discrimination within the sampled A/B contrast. Uncertainty intervals and Youden cut-points were quantified by resampling complete field pairs with replacement (1000 pair-bootstrap replicates; Table 4). Pair-preserving LOPO cross-validation was used separately to assess internal cut-point classification. These diagnostic analyses do not represent physiological salt-tolerance thresholds or externally validated diagnostic performance.

**Figure 5:**
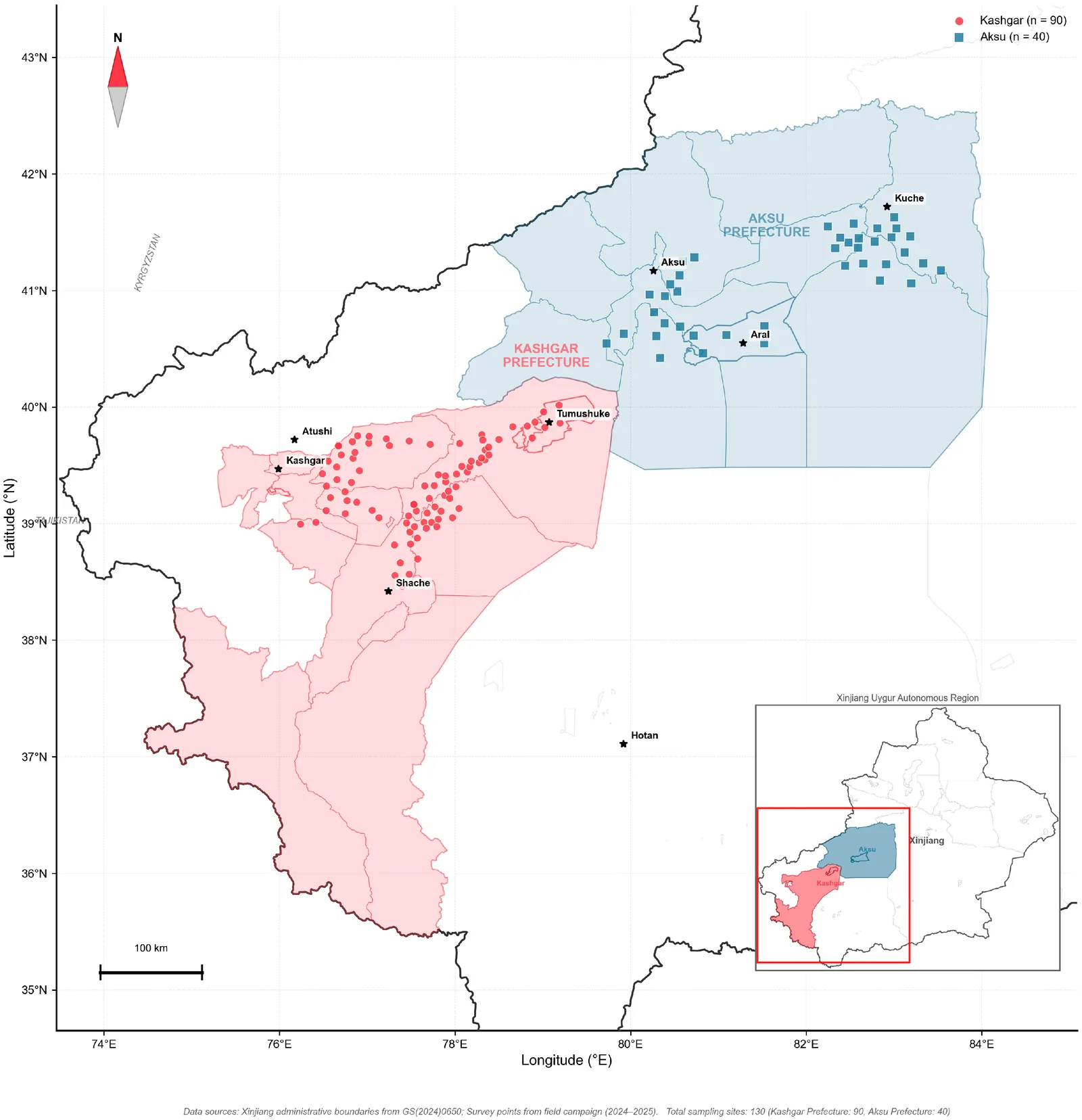
Study area and paired sampling locations in southern Xinjiang, China. Kashgar contributed 90 complete field pairs and Aksu 40 complete field pairs. Each field pair consisted of one well-established A microsite (live-seedling stand ≥ 80%) and one nearby non-emerged or nearly non-emerged B microsite (emergence < 5%) within the same commercial field. Administrative boundaries are shown only to indicate the study area and do not imply any position regarding legal status or delimitation.

**Table 1:** Paired comparison of 0–10 cm seed-zone soil properties between emerged (A) and non-emerged (B) microsites across 130 fields.

Variable	Unit	A Mean ± SD	B Mean ± SD	HL (B − A)	B > A (%)	FDR q
pH	—	8.30 ± 0.23	8.44 ± 0.28	0.15	66.9	<0.001
Total salt	g kg^−1^	5.95 ± 4.19	13.54 ± 7.00	7.00	95.4	<0.001
EC_1:5_	mS cm^−1^	1.21 ± 0.75	2.98 ± 1.37	1.66	97.7	<0.001
HCO_3_^−^	g kg^−1^	0.35 ± 0.07	0.38 ± 0.10	0.03	42.3	<0.001
Cl^−^	g kg^−1^	0.46 ± 0.35	2.17 ± 1.79	1.46	93.8	<0.001
SO_4_^2−^	g kg^−1^	2.88 ± 2.30	5.54 ± 2.17	2.58	92.3	<0.001
Ca^2+^	g kg^−1^	1.18 ± 1.08	1.88 ± 0.86	0.66	70.0	<0.001
Mg^2+^	g kg^−1^	0.08 ± 0.05	0.30 ± 0.31	0.17	92.3	<0.001
K^+^	g kg^−1^	0.06 ± 0.03	0.11 ± 0.08	0.04	86.2	<0.001
Na^+^	g kg^−1^	0.28 ± 0.18	1.54 ± 1.41	1.01	95.4	<0.001
SAR_1:5_	—	1.32 ± 1.14	4.17 ± 3.36	2.43	86.9	<0.001
Na^+^/(Ca^2+^ + Mg^2+^)	—	0.60 ± 0.78	0.98 ± 1.55	0.31	66.2	<0.001
Cl^−^/SO_4_^2−^	—	0.36 ± 0.49	0.39 ± 0.27	0.11	65.4	0.004
Clay	%	9.01 ± 6.25	6.60 ± 4.78	−2.10	35.4	<0.001
Silt	%	22.70 ± 12.62	15.08 ± 12.06	−7.50	30.0	<0.001
Fine sand	%	60.76 ± 15.10	73.06 ± 13.97	12.30	73.1	<0.001
Coarse sand	%	7.54 ± 9.37	5.25 ± 4.67	−0.55	54.6	0.313

Note: HL = Hodges–Lehmann estimator of the paired B − A difference. Negative values indicate higher A than B. q values are Benjamini–Hochberg-adjusted Wilcoxon signed-rank *p* values. For HCO_3_^−^, 34.6% of pairs were exact ties; therefore, the proportion B > A should not be interpreted as a sign test. The Wilcoxon signed-rank test and Hodges–Lehmann estimator incorporate the magnitudes and ranks of non-zero paired differences.

**Table 2:** Matched conditional logistic regression for the non-emerged microsite (B), with field pair as the matched stratum.

Model	Predictor	OR	95% CI	*p*
M1: EC + texture	ln(EC_1:5_ + 1), standardized	234.99	10.88–5073.50	<0.001
M1: EC + texture	Clay, standardized	1.04	0.32–3.38	0.945
M1: EC + texture	Silt, standardized	1.66	0.49–5.61	0.414
M2: EC + Na + texture	ln(EC_1:5_ + 1), standardized	41.30	2.05–831.30	0.015
M2: EC + Na + texture	ln(Na^+^ + 1), standardized	7.57	0.72–79.57	0.092
M2: EC + Na + texture	Clay, standardized	1.09	0.31–3.80	0.890
M2: EC + Na + texture	Silt, standardized	1.76	0.51–6.00	0.370
M3: EC + Cl + texture	ln(EC_1:5_ + 1), standardized	47.16	2.40–926.27	0.011
M3: EC + Cl + texture	ln(Cl^−^ + 1), standardized	5.34	0.80–35.48	0.083
M3: EC + Cl + texture	Clay, standardized	1.15	0.32–4.18	0.831
M3: EC + Cl + texture	Silt, standardized	1.73	0.55–5.49	0.349
M4: M2 + Na × region	ln(EC_1:5_ + 1), standardized	37.08	1.51–910.14	0.027
M4: M2 + Na × region	ln(Na^+^ + 1), standardized	5.84	0.12–286.28	0.374
M4: M2 + Na × region	Na^+^ × Kashgar interaction	1.50	0.01–189.05	0.870

Note: ORs are reported per 1-SD increase in transformed/standardized predictors. The region interaction uses Aksu as the reference region. The analogous EC_1:5_ × region model was not used for inference because near-perfect EC discrimination in Aksu produced quasi-complete separation.

**Table 3:** Paired tests of marginal homogeneity for three predefined classifications: salinity severity (total water-soluble salt classes), salt chemical type (Cl^−^/SO_4_^2−^ ratio classes), and pH class.

Classification	Stuart–Maxwell χ^2^	df	*p*
Salinity severity	84.50	4	<0.001
Salt chemical type	6.83	2	0.033
pH class	10.57	2	0.005

**Table 4:** Pair-bootstrap ROC performance and exploratory diagnostic cut-points for key seed-zone indicators.

Region	Indicator	AUC	95% CI	Cut-Point	Cut-Point 95% CI	Sens.	Spec.
Combined	Na^+^ (g kg^−1^)	0.909	0.875–0.942	0.546	0.422–0.728	0.800	0.915
Combined	EC_1:5_ (mS cm^−1^)	0.902	0.877–0.928	2.23	1.42–2.51	0.746	0.877
Combined	Cl^−^ (g kg^−1^)	0.892	0.856–0.927	0.803	0.502–1.15	0.769	0.900
Combined	Total salt (g kg^−1^)	0.837	0.806–0.869	7	5–9.2	0.892	0.662
Combined	SAR_1:5_	0.815	0.763–0.868	1.89	1.28–2.62	0.754	0.785
Kashgar	Na^+^ (g kg^−1^)	0.944	0.906–0.975	0.546	0.46–0.956	0.867	0.911
Kashgar	EC_1:5_ (mS cm^−1^)	0.898	0.861–0.936	2.51	2.23–2.61	0.722	0.967
Kashgar	Cl^−^ (g kg^−1^)	0.953	0.919–0.978	1.21	0.924–1.42	0.856	0.944
Kashgar	Total salt (g kg^−1^)	0.842	0.801–0.880	13.7	9.9–13.9	0.567	0.967
Kashgar	SAR_1:5_	0.902	0.859–0.941	2.14	2.08–2.72	0.833	0.867
Aksu	Na^+^ (g kg^−1^)	0.865	0.776–0.943	0.329	0.254–0.632	0.775	0.875
Aksu	EC_1:5_ (mS cm^−1^)	0.999	0.994–1.000	1.17	1.17–1.42	1.000	0.975
Aksu	Cl^−^ (g kg^−1^)	0.858	0.781–0.933	0.502	0.323–0.803	0.750	0.875
Aksu	Total salt (g kg^−1^)	0.999	0.995–1.000	5	4.7–5.7	0.975	1.000
Aksu	SAR_1:5_	0.597	0.472–0.717	1.89	1.24–2.12	0.525	0.775

Note: Complete A–B field pairs were resampled with replacement (1000 replicates). Cut-points maximize Youden J in the sampled contrast and should be interpreted as exploratory field diagnostic cut-points rather than physiological thresholds. Values in Table 4 are apparent full-sample performance; pair-preserving leave-one-pair-out cross-validation was used as an internal validation of cut-point classification and is reported in Section 2.5. Neither procedure constitutes external validation.

## Data Availability

The field-level dataset is not publicly available because it was collected from commercial farmers’ fields under data-use arrangements that did not include public redistribution. Although geographic coordinates and field identifiers could be removed, combinations of site-level information, including study region, cultivar, sowing date, crop-emergence and salt-damage observations, and management information, could still permit indirect identification of individual fields or participating growers. Aggregate results and all information required to reproduce the reported analyses are provided in the manuscript.
